# Mechanisms of action of Zishen Yutai pills in treating premature ovarian failure determined by integrating UHPLC-Q-TOF-MS and network pharmacology analysis

**DOI:** 10.1186/s12906-022-03763-2

**Published:** 2022-10-26

**Authors:** Lei Dang, Chunbo Zhang, Biru Su, Na Ning, Qiuling Huang, Su Zhou, Meng Wu, Wenqing Ma, Man Wang, Pengfei Cui, Yan Li, Shixuan Wang

**Affiliations:** 1 Department of Obstetrics and Gynecology, Tongji Hospital, Tongji Medical College, Huazhong University of Science and Technology, Wuhan, Hubei China; 2 Post-Doctoral Research Center of Guangzhou Pharmaceutical Holdings Ltd, Guangzhou, Guangdong China; 3 Guangzhou Baiyunshan Zhongyi Pharmaceutical Co. Ltd, Guangzhou, Guangdong China

**Keywords:** Active compound, Molecular mechanism, Network pharmacology, Premature ovarian failure, Therapeutic mechanism, Zishen Yutai pills

## Abstract

**Background:**

Zishen Yutai (ZSYT) pill, a patent Chinese medicine, has been widely used in the treatment of infertility, abortion, and adjunctive treatment of in vitro fertilization (IVF) for decades. Recently, the results of clinical observations showed that premature ovarian failure (POF) patients exhibited improved expression of steroids and clinical symptoms associated with hormone disorders after treatment with Zishen Yutai pills. However, the pharmacological mechanism of action of these pills remains unclear.

**Methods:**

The compounds of Zishen Yutai pills found in blood circulation were identified via ultra-high performance liquid chromatography-quadrupole time-of-flight mass spectrometry (UHPLC-Q-TOF-MS) technique in the serum of POF mice after oral administration of Zishen Yutai pills. The potential targets of compounds were screened using Traditional Chinese Medicine Systems Pharmacology Database, Traditional Chinese Medicine Database@Taiwan, Drugbank Database, PubChem, HIT, Pharmapper, and Swiss Target Prediction. The target genes associated with POF were collected from Online Mendelian Inheritance in Man Database, PharmGkb, Genecards, Therapeutic Target Database, and Genetic Association Database. The overlapping genes between the potential targets of Zishen Yutai pills’ compounds and the target genes associated with POF were clarified via protein-protein interaction (PPI), pathway, and network analysis.

**Results:**

Nineteen compounds in Zishen Yutai pills were detected in the serum of POF mice after oral administration. A total of 695 Zishen Yutai (ZSYT) pill-related targets were screened, and 344 POF-related targets were collected. From the results of Zishen Yutai (ZSYT) pill-POF PPI analysis, CYP19A1, AKR1C3, ESR1, AR, and SRD5A2 were identified as key targets via network analysis, indicating their core role in the treatment of POF with Zishen Yutai pills. Moreover, the pathway enrichment results suggested that Zishen Yutai pills treated POF primarily by regulating neuroactive ligand-receptor interaction, steroid hormone biosynthesis, and ovarian steroidogenesis.

**Conclusions:**

Via virtual screening, we found that regulation of neuroactive ligand-receptor interaction, steroid hormone biosynthesis, and ovarian steroidogenesis was the potential therapeutic mechanism of Zishen Yutai pills in treating POF. Our study suggested that combining the analysis of Zishen Yutai pills’ compounds in blood in vivo in the POF model and network pharmacology prediction might offer a tool to characterize the mechanism of Zishen Yutai pills in the POF.

**Supplementary Information:**

The online version contains supplementary material available at 10.1186/s12906-022-03763-2.

## Background

Premature ovarian failure (POF) is an ovarian dysfunction with cessation of menstruation before 40 years of age [1]. POF probably occurs when the number of ovarian follicles is exhausted before the typical age of physiological menopause [2, 3].

The pathological causes of POF include genetic, autoimmune, therapeutic, and environmental stimulus, etc. [4]. The prevalence of POF in women is approximately 1% [5], and it occurs in women between 20 and 40 years of age [6].

The clinical diagnostic criteria of POF is amenorrhea together with elevated gonadotropin levels (follicle-stimulating hormone [FSH] > 20 IU/L), low estradiol (E2) levels (< 20 pg/mL), and low anti-Müllerian hormone (AMH) levels –< 0.5 ng/mL (< 1 ng/mL) [7]. POF affects the quality of life of women by causing hot flushes, excessive sweating, hair loss, skin and mucous membrane dryness, and even loss of fertility that is devastating for women of reproductive age [1]. In the long term, women with POF face an increased risk of morbidity, developing conditions such as osteoporosis, cardiovascular diseases, and type 2 diabetes. Because of the complexity of POF pathogenesis, the optimal remedy for the treatment of POF is still under investigation [4].

Traditional Chinese medicine (TCM) has been widely used in treating gynecological disorders in Asian countries and has been proposed for treating POF [8]. Zishen Yutai (ZSYT) pills, a Chinese patent medicine, have been clinically used to treat infertility, abortion, and adjunctive treatment of in vitro fertilization (IVF) [9, 10]. Recent studies with Zishen Yutai pills identified the improvement in clinical symptoms of hormone disorders and the increase in the FSH and luteinizing hormone (LH) levels in the treatment group compared to the controls, indicating the clinical potential of Zishen Yutai pills in treating POF [11, 12]. Although the methanol extract of Zishen Yutai pills was analyzed previously in vitro via UHPLC-Q-TOF-MS [13], the active compounds in vivo and their pharmacological targets in treating POF have not been elucidated. Our study was designed to explore the synergistic mechanism of absorbed compounds in vivo from Zishen Yutai pills in treating POF.

Zishen Yutai pills contained 15 herbal materials. The complexity of herbal materials made a difficulty in studying the synergistic effects of TCM prescriptions. The research strategy of UHPLC-Q-TOF-MS technology in analyzing the plentiful absorbed compounds in combination with network pharmacology via virtual screening has made the mission possible [14].

**Fig. 1:**
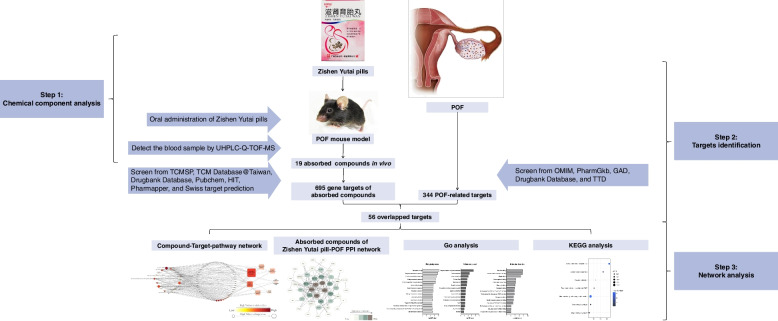
Schematics of the process for identifying absorbed compounds of Zishen Yutai pills in the POF mouse serum and determining their targets of activity against POF via integrated UHPLC-Q-TOF-MS analysis and network pharmacology approach

In this study, the UHPLC-Q-TOF-MS analysis approach was used to identify the absorbed compounds of the Zishen Yutai pills in the POF mouse model in vivo. Next, network pharmacology analysis was performed to screen potential active ingredients, seek their targets for treating POF, and investigate the underlying pharmacological mechanisms. The combination of UHPLC-Q-TOF-MS and network pharmacology helped to build the pharmacology network for exploring the therapeutic mechanism of Zishen Yutai pills in the treatment of POF, and might lead to a full understanding of the interactions taking place between absorbed compounds of Zishen Yutai pills in vivo and potential targets involved in the POF treatment (Fig. 1).

## Methods

### Preparation of Zishen Yutai pills

Fifteen herbs, *Semen Cuscutae*, *Fructus Amomi Villosi*, *Radix Rehmanniae Preparata*, *Radix Ginseng*, *Herba Taxilli, Equus asinus L.*, *Radix Polygoni Multiflori*, *Folium Artemisiae Argyi*, *Radix Morindae Officinalis*, *Rhizoma Atractylodis Macrocephalae*, *Radix Codonopsis*, *Cornu Cervi Degelatinatum*, *Fructus Lycii*, *Radix Dipsaci*, and *Cortex Eucommiae*, comprising the Zishen Yutai pills (batch No. A00010) were provided by Guangzhou Baiyunshan Zhongyi Pharmaceutical Co., Ltd. The main ingredient of *Cornu Cervi Degelatinatum* was Ca_3_(PO_4_)_2_ and it has no botanical name [15]. Botanical materials were authenticated before manufacturing in good manufacturing practice–certified pharmaceutical factories. The preparation and quality control of the Zishen Yutai pills were performed according to the Chinese Pharmacopeia (2020 edition) and the Ministry of Public Health of the People’s Republic of China – TCM prescription preparation (Volume 16, standard number: WS_3_-B-3113-98). For the experiments, Zishen Yutai pills were broken into powder, passed through an 80-mesh screen, and stirred uniformly in pilot facilities.

### Animals and treatment

Female C57BL/6 mice between 6 and 8 weeks of age were obtained from the HUNAN SJA Laboratories Animal Co., Ltd. The mice were housed in a temperature-controlled environment (22 ± 2 °C) under a 12/12 h light/dark cycle with a relative humidity of 60%. The mice were separated into five mice per cage for seven days to adapt to the environment. All mice were provided free access to chow and tap water.

After one-week acclimatization, 20 C57BL/6 female mice were randomly divided into two groups: POF and POF + Zishen Yutai (ZSYT) pill, with 10 mice in each group. Mice in the POF and POF + Zishen Yutai (ZSYT) pill groups were treated with cisplatin to induce POF. The mice were intraperitoneally administered 2 mg/kg cisplatin (Sigma Aldrich, USA) once a day for 10 days [16, 17]. Cisplatin was freshly prepared in saline immediately before use.

After intraperitoneal administration for 10 days, the mice in the POF + Zishen Yutai (ZSYT) pill group were orally administered with Zishen Yutai pills (3.90 g·kg^− 1^ body weight), whereas the mice in the POF group were orally administered with an equal volume of 0.3% CMC-Na solution. The Zishen Yutai pills’ powder was dissolved in 0.3% CMC-Na solution as an oral suspension. All mice were dosed twice daily for 3 days [18]. All mice were euthanized to collect blood samples 1 h after the last dose.

The experiments and procedures were performed following the principles of laboratory animal use and care. The study was approved by the Institutional Animal Ethics Committee of the Guangzhou General Pharmaceutical Research Institute Co., Ltd.

### Collection and preparation of serum samples

Blood samples were collected from the abdominal aorta after euthanasia. Blood samples were left to stand at room temperature for 30 min and then centrifuged at 3000 rpm for 10 min at 4 °C to obtain serum. The serum supernatants were collected and mixed at equal volumes before storage at − 80 °C. Next, the 100 μL of the serum mixture was mixed with 400 μL of methanol, whirled for 3 min, and then centrifuged at 13,000 rpm for 10 min at 4 °C. The 300 μL of supernatant was transferred to a clean tube and dried under a gentle flow of nitrogen gas at 30 °C. The residue was dissolved in 75 μL of methanol and centrifuged at 13000 rpm for 10 min at 4 °C [19]. The supernatant was collected, and 4 μL was injected for UHPLC-Q-TOF-MS analysis.

### Chromatography conditions and UHPLC-Q-TOF-MS analysis

The UHPLC chromatographic qualitative analysis was performed on a SCIEX X500R QTOF LC-MS/MS system (AB SCIEX Pte Ltd., USA), equipped with an ExionLC degasser, an ExionLC AD pump, an ExionLC AD autosampler, an ExionLC AD column oven, and an ExionLC PDA detector.

A Hypersil gold C18 (150 mm × 2.1 mm, 1.9 μm, Thermo Fisher) column was used to separate the extract components. Mobile phase A consisted of 0.1% formic acid (v/v) solution, and mobile phase B was acetonitrile. The flow rate was 0.4 mL min^− 1^ and the column temperature was maintained at 40 °C. The gradient program was set as follows: 0–10 min, 5% B; 10-20 min, 5–10% B; 20 − 40 min, 10–25% B; 40 − 60 min, 25–30% B; 60 − 75 min, 30–40% B; 75 − 90 min, 40–75% B; 90 − 120 min, 75 − 90% B; 120 − 140 min, 90 − 100% B; 140 − 190 min, 100% B [13]. Data acquisition and analysis were carried out using Sciex PeakView™ 1.7 software.

### Mass spectrometry

Mass spectrometry of serum samples was conducted on a SCIEX X500R QTOF mass spectrometer (AB SCIEX Pte Ltd., USA) equipped with an electrospray ionization (ESI) source connected to the UPLC system. Sciex PeakView™ 1.7. software (AB SCIEX Pte Ltd., USA) was used for data acquisition and processing.

The analysis was performed in both positive and negative ESI ion modes. The information-dependent acquisition scan mode was selected, and the mass range was recorded at m/z 100–1500 [13]. Calibration solutions for the SCIEX X500 System were used to calibrate the mass spectrometer in the positive and negative ion modes.

### Data processing

After data acquisition via UHPLC-Q-TOF-MS, compounds simultaneously with the same mass and equal fragment intensity between the results from the POF + Zishen Yutai (ZSYT) pill and POF groups were excluded. Next, the mass and fragments of the remaining candidate compounds were matched with our previously detected compounds from methanol extract of Zishen Yutai pills in vitro [13] and online databases including the Chemspider (http://www.chemspider.com/), SCIEX-Standard Chinese medicine Mass Spectral Database, SCIEX metabolite Mass Spectral Database, and SCIEX lipids Mass Spectral Database [19, 20]. Thereafter, the Zishen Yutai pills’ compounds found in the POF mouse in vivo were identified.

### Target collection

The relevant targets of the Zishen Yutai pills’ compounds identified in vivo were obtained from the Traditional Chinese Medicine Systems Pharmacology Database (TCMSP, http://lsp.nwu.edu.cn/tcmsp.php), TCM Database@Taiwan (http://tcm.cmu.edu.tw/), Drugbank Database (https://www.drugbank.ca/), PubChem (https://pubchem.ncbi.nlm.nih.gov/), HIT (lifecenter. Sgst. cn/hit /), Pharmapper (http://www.lilab-ecust.cn/pharmmapper/), and Swiss target prediction (http://www.swisstargetprediction.ch/) [21]. By inputting all molecular formulae of the absorbed compounds into these databases, we obtained information on their potential targets; sufficient targets associated with the absorbed compounds were obtained.

The target genes associated with POF were collected from the Online Mendelian Inheritance in Man Database (OMIM, http://www.omim.org/), Pharmacogenomics Knowledge Base (PharmGkb, https://www.pharmgkb.org/), Genecards (http://www.genecards.org/), Therapeutic Target Database (TTD, https://db.idrblab.org/ttd/), and Genetic Association Database (GAD, https://geneticassociationdb.nih.gov/).

POF-related targets overlapping with the absorbed compound-relevant targets of Zishen Yutai pills were retained for network construction and analysis.

### Network analysis and construction

To explore the Zishen Yutai pills’ mechanisms of action in the treatment of POF, we investigated the pathways associated with POF treatment. First, the STRING database (http://string-db.org) was used to filter possible protein-protein interactions (PPIs) [22]. The attained PPIs with high confidence > 0.7 were selected for the following analysis. The core targets were selected in accordance to the degree in the PPIs results. Second, the shared targets between the Zishen Yutai pills’ compounds in vivo and POF were constructed and their modulated pathway were identified through Kyoto Encyclopedia of Genes and Genomes (KEGG) database [23]. Then, the absorbed compounds of Zishen Yutai pills in vivo, shared targets between the compounds of Zishen Yutai pills in vivo and POF, and their related pathway were constructed network to visualize using Cytoscape v3.6.1. The absorbed compounds of Zishen Yutai pills, target genes, and related pathway were expressed as nodes, whereas the interactions among compounds, potential targets, and related pathway were expressed as edges in the graphical networks. The node size and colors in the network were set as “*high values to large size*” and “*high values to dark colors*” respectively on basis of the edge count for both settings [24, 25]. Thirdly, the Go enrichment analysis gene ontology (GO) and KEGG enrichment were conducted using the functional annotation tool of DAVID Bioinformatics Resources 6.7 (http://david.abcc.ncifcri.gov/) and Bioinformatics (http://www.bioinformatics.com.cn/), respectively [26–29] .

## Results

### Screening for the absorbed compounds of Zishen Yutai pills in vivo

**Fig. 2:**
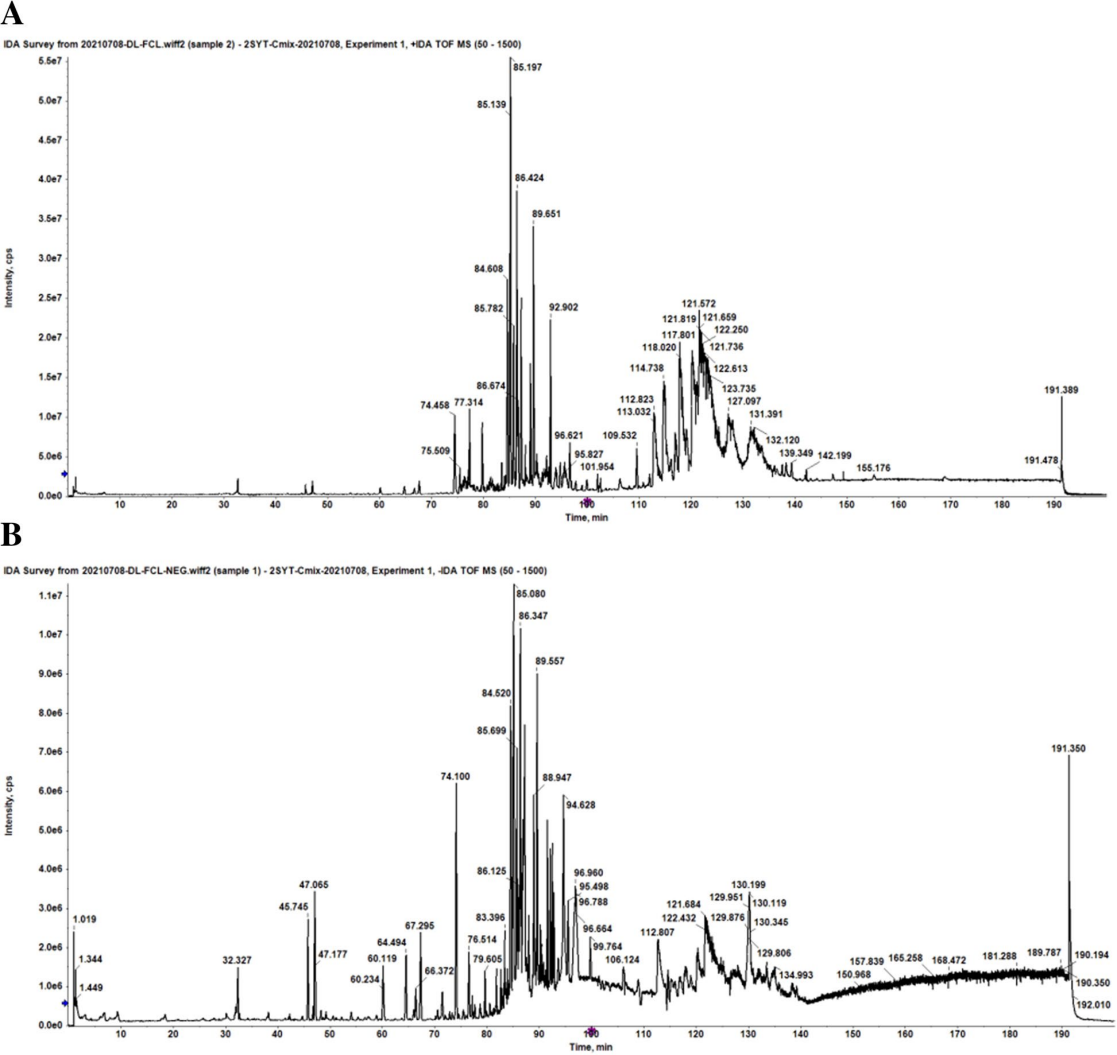
Total ion flow diagram of the absorbed compounds in serum after the oral administration of Zishen Yutai pills in POF mice analyzed via UHPLC-Q-TOF-MS. **A** Positive ion mode. **B** Negative ion mode

The medicated serum samples were collected after the oral administration of Zishen Yutai pills in the POF mice, while the blank serum samples were collected after the oral administration of 0.3% CMC-Na solution in the POF mice. The both samples were analyzed via UHPLC-Q-TOF-MS. The peaks of candidate compounds in the medicated serum samples were extracted from both positive and negative ion chromatograms after excluding the same mass with equal fragment intensity in the blank serum **(**Fig. 2**)**. The candidate compounds were identified qualitatively using Sciex PeakView™ 1.7. software [30].

The MS/MS spectra of all candidate compounds were evaluated and matched with hits in the Chemspider (http://www.chemspider.com/), SCIEX-Standard Chinese medicine Mass Spectral Database, SCIEX metabolite Mass Spectral Database, SCIEX lipids Mass Spectral Database, and our previously identified chemical profiles of Zishen Yutai pills in vitro.

A total of 19 compounds were successfully characterized by the comparison with the online database and our previously identified compounds (Table 1). 6 compounds were identified previously, and 13 compounds were newly identified.

**Table 1:** Absorbed compounds in serum after the oral administration of Zishen Yutai pills in POF mice

**No.**	**Identification**	**Ionization mode**	**Retention Time (t/min)**	**Observed m/z (+/-)**	**formula**	**Molecular Structure**
M1	3,5-Pyridinedicarboxylic acid, 1,4-dihydro-2,6-dimethyl-4-[3-[4-(4-morpholinylsulfonyl)phenyl]-1-phenyl-1H-pyrazol-4-yl]-, 3,5-bis(2-methylpropyl) ester	[M+H]+	95.91	677.3005	C_36_H_44_N_4_O_7_S	
M2	Trichosanic acid	[M+H]+	90.55	279.2323	C_18_H_30_O_2_	
M3	linolenic acid	[M+H]+	90.55	279.2323	C_18_H_30_O_2_	
M4	Tetradecyl (βS)-β-hydroxy-N-(phenylacetyl)-D-phenylalaninate	[M+H]+	86.52	496.3404	C_31_H_45_NO_4_	
M5	Vernolic acid	[M+H]+	80.74	297.2426	C_18_H_32_O_3_	
M6	cholic acid	[M+NH4]+	74.45	426.322	C_24_H_40_O_5_	
M7	3-(2-Furylmethyl)-10-methyl-2-(3-nitrophenyl)pyrimido[4,5-b]quinoline-4,5(3H,10H)-dione	[M+Na]+	74.16	451.1004	C_23_H_16_N_4_O_5_	
M8	1-[(2R,4aS,4bS,6aS,6bS,9aR,10aS,10bR,12aS)-2-Hydroxy-4a,6a,8,8-tetramethylhexadecahydro-6bH-naphtho[2',1':4,5]indeno[1,2-d][1,3]dioxol-6b-yl]ethanone	[M+H]+	67.63	391.2838	C_24_H_38_O_4_	
M9	Ethyl N-[(13E,15R)-15-hydroxy-1,9-dioxoprost-13-en-1-yl]glycinate	[M+H]+	66.69	424.3053	C_24_H_41_NO_5_	
M10	(3α,9ξ,14ξ,16α)-17-Hydroxy-16-methyl-20-oxopregn-5-en-3-yl acetate	[M+H]+	66.69	389.268	C_24_H_36_O_4_	
M11	(3β,5α)-16-Methyl-20-oxopregn-16-en-3-yl acetate	[M+H]+	64.75	373.2731	C_24_H_36_O_3_	
M12	Corticosterone	[M+H]+	52.49	347.221	C_21_H_30_O_4_	
M13	Cortisol	[M+H]+	48.43	363.2163	C_21_H_30_O_5_	
M14	2-[[(3Î±,5Î²)-3-Hydroxy-7,24-dioxocholan-24-yl]amino]ethanesulfonic acid	[M+H]+	47.04	498.2884	C_26_H_43_NO_6_S	
M15	Taurocholic acid	[M+H]+	45.73	516.2987	C_26_H_45_NO_7_S	
M16	Benzophenone	[M+H]+	16.37	183.0804	C_13_H_10_O	
M17	L-Pyroglutmaic acid	[M+H]+	1.46	130.0498	C_5_H_7_NO_3_	
M18	Isoleucine	[M+H]+	1.04	132.1017	C_6_H_13_NO_2_	
M19	Betaine	[M+H]+	1.03	118.0863	C_5_H_11_NO_2_	

### Shared targets between absorbed compound-related targets of Zishen Yutai pills in vivo and POF-related targets

Based on the results of UHPLC-Q-TOF-MS analysis, 19 absorbed compounds of Zishen Yutai pills in vivo were used to obtain the Zishen Yutai (ZSYT) pill-related targets. In this study, the 695 Zishen Yutai (ZSYT) pill-related targets were screened from the TCMSP, TCM Database@Taiwan, Drugbank Database, PubChem, HIT, Pharmapper, and Swiss target prediction corresponding to the 19 compounds from the Zishen Yutai pills absorbed in vivo.

The POF-related target proteins were screened from OMIM, Pharmacogenomics Knowledge base (PharmGkb), GAD, Drugbank Database, and TTD. Only “*Homo sapiens*” proteins linked to POF were selected. We obtained a total of 344 genes.

**Fig. 3:**
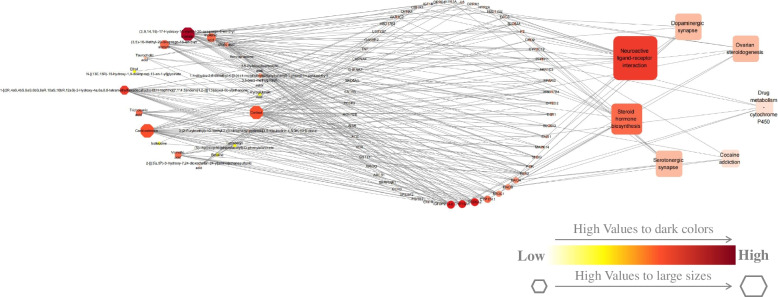
The compound-target-pathway network for the absorbed compounds of Zishen Yutai pills by orally administered in a POF mouse model

Among all targets from Zishen Yutai pills and POF, 56 overlapped genes were identified **(**Table 2**)**. According to the overlapped genes, a total of 7 modulated pathways were found to be regulated by Zishen Yutai pills in the POF treatment. Both overlapped genes and modulated pathways were retained for network construction **(**Fig. 3**)**. The composed compound-target-pathway network consisted of 82 nodes and 229 edges which included 51 edges for pathway-target interactions and 178 edges for compound-target interactions (Table S1).

**Table 2:** POF-related target genes interacting with absorbed compounds from Zishen Yutai pills

No.	Symbol Name
**1**	ABCB1
**2**	ACE
**3**	ACVR2B
**4**	AKR1C2
**5**	AKR1C3
**6**	APBA3
**7**	AR
**8**	CCR3
**9**	CHFR
**10**	CHRNA4
**11**	CHRNA7
**12**	CITED2
**13**	CNR1
**14**	CRHR1
**15**	CYP2C19
**16**	CYP17A1
**17**	CYP19A1
**18**	DRD2
**19**	DRD3
**20**	ESR1
**21**	ESR2
**22**	FGFR1
**23**	F2
**24**	GABBR2
**25**	GSTT1
**26**	HSD17B2
**27**	HSD17B3
**28**	HSD17B4
**29**	HTR1A
**30**	HTR2A
**31**	IGFBP2
**32**	IGF1R
**33**	IL6
**34**	INSR
**35**	MAOA
**36**	MAOB
**37**	MAPK14
**38**	NR3C1
**39**	OPRD1
**40**	OPRK1
**41**	OPRM1
**42**	PCSK9
**43**	PGR
**44**	PPARG
**45**	PTPN11
**46**	SERPINE1
**47**	SHBG
**48**	SLC6A3
**49**	SLC6A4
**50**	SRD5A1
**51**	SRD5A2
**52**	SREBF2
**53**	SSTR3
**54**	TNF
**55**	UGT2B7
**56**	VDR

### Absorbed compounds of Zishen Yutai (ZSYT) pill-POF PPI network

A total of 56 overlapping genes were obtained by searching for the intersection of the targets of absorbed compounds from Zishen Yutai pills in vivo and the 344 POF-related targets.

Among the overlapping genes, a total of 202 PPIs with high confidence (*P*-value > 0.7) were obtained and selected from STRING databases for PPI network construction using Cytoscape software (Table S2).

**Fig. 4:**
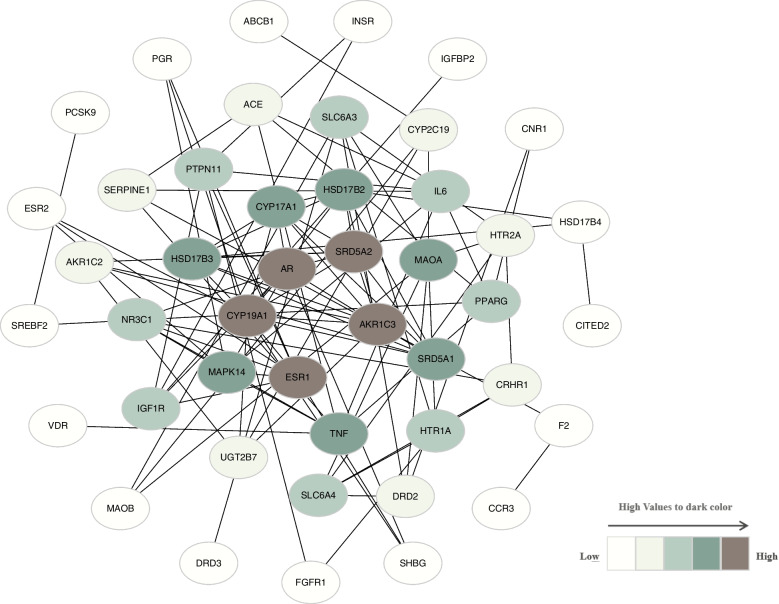
The absorbed compounds of Zishen Yutai pill-POF PPI network. The node color in the network were set as “*high values to dark colors*” on basis of the degree

The absorbed compounds of the Zishen Yutai (ZSYT) pill-POF PPI network formed 45 nodes and 100 edges (Fig. 4). The key targets were analyzed using central network evaluation. Twelve targets with 50 interactions were found with a degree greater than 6 (Table S3) after analysis via Cytohubba. Among the top-ranked hub targets, CYP19A1, aldo-keto reductase (AKR) 1C3, ESR1, AR, and SRD5A2 were identified as key targets via network analysis, indicating their core role in the treatment of POF by Zishen Yutai pills.

### Pathway enrichment

**Fig. 5:**
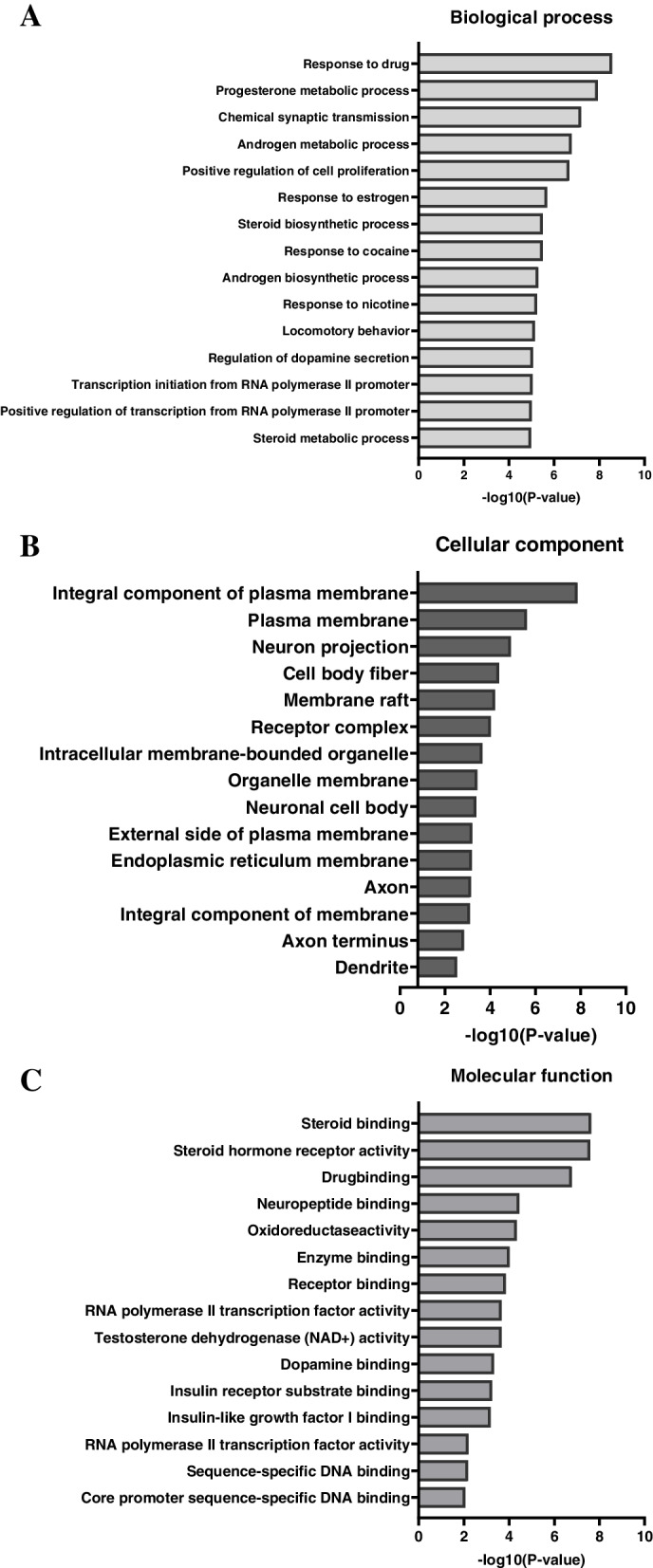
GO enrichment analysis of therapeutic targets of orally absorbed compounds from Zishen Yutai pills in POF. **A** Biological process. **B** Cellular components. **C** Molecular functions

GO enrichment analysis showed that the majority of these 56 targets were identified using the DAVID Bioinformatics Resources. There were 183 biological processes (BP), 24 cellular components (CC), and 62 molecular functions (MF) identified. The top 15 significantly enriched terms in the BP, CC, and MF categories (*P* < 0.05) were shown in Fig. 5A-C. Specifically, most of these target proteins were enriched in responses to drugs, progesterone, androgen metabolic processes, chemical synaptic transmission, positive regulation of cell proliferation, and steroid and androgen biosynthetic processes, indicating multiple synergies of absorbed compounds from Zishen Yutai pills with biological processes. The most of regulated pathways in the BP like progesterone metabolic processes, androgen metabolic process, response to estrogen, steroid biosynthetic process, androgen biosynthetic process, and steroid metabolic process directly and indirectly involved in the biological estrogen synthesis and female reproductive regulation. And the response to estrogen and steroid biosynthetic processes were primarily regulated by triggering 6 genes (*CITED2*, *SRD5A1*, *IGFBP2*, *OPRK1*, *PPARG*, *ESR1*) and 5 genes (*SRD5A2*, *SRD5A1*, *HSD17B2*, *CYP19A1*, *CYP17A1*) at the false discovery rate of 3.32E-04 and 3.94E-04, respectively. It also revealed that the absorbed compounds from Zishen Yutai pills might influence the steroid binding, steroid hormone receptor activity, and drug binding in MF. The steroid binding and steroid hormone receptor activity were identified as main regulation pathways in MF at the false discovery rate of 3.49E-06 and 3.49E-06, which influenced 6 genes (*AR*, *PGR*, *NR3C1*, *SHBG*, *ESR1*, *ESR2*) and 7 genes (*AR*, *VDR*, *PGR*, *PPARG*, *NR3C1*, *ESR1*, *ESR2*), respectively. Furthermore, the integral compound of plasma membrane, or plasma membrane at the false discovery rate of 7.72E-06 and 6.64E-04 in CC potentially involved in the therapeutic effect on POF via concurrently regulating *ACVR2B*, *CCR3*, *CNR1*, *CRHR1*, *DRD2*, *DRD3*, *FGFR1*, *GABBR2*, *HTR1A*, *HTR2A*, *IGF1R*, *INSR*, *OPRD1*, *OPRK1*, *OPRM1*, *SLC6A3*, *SLC6A4*, *SSTR3*, and *TNF*.

**Fig. 6:**
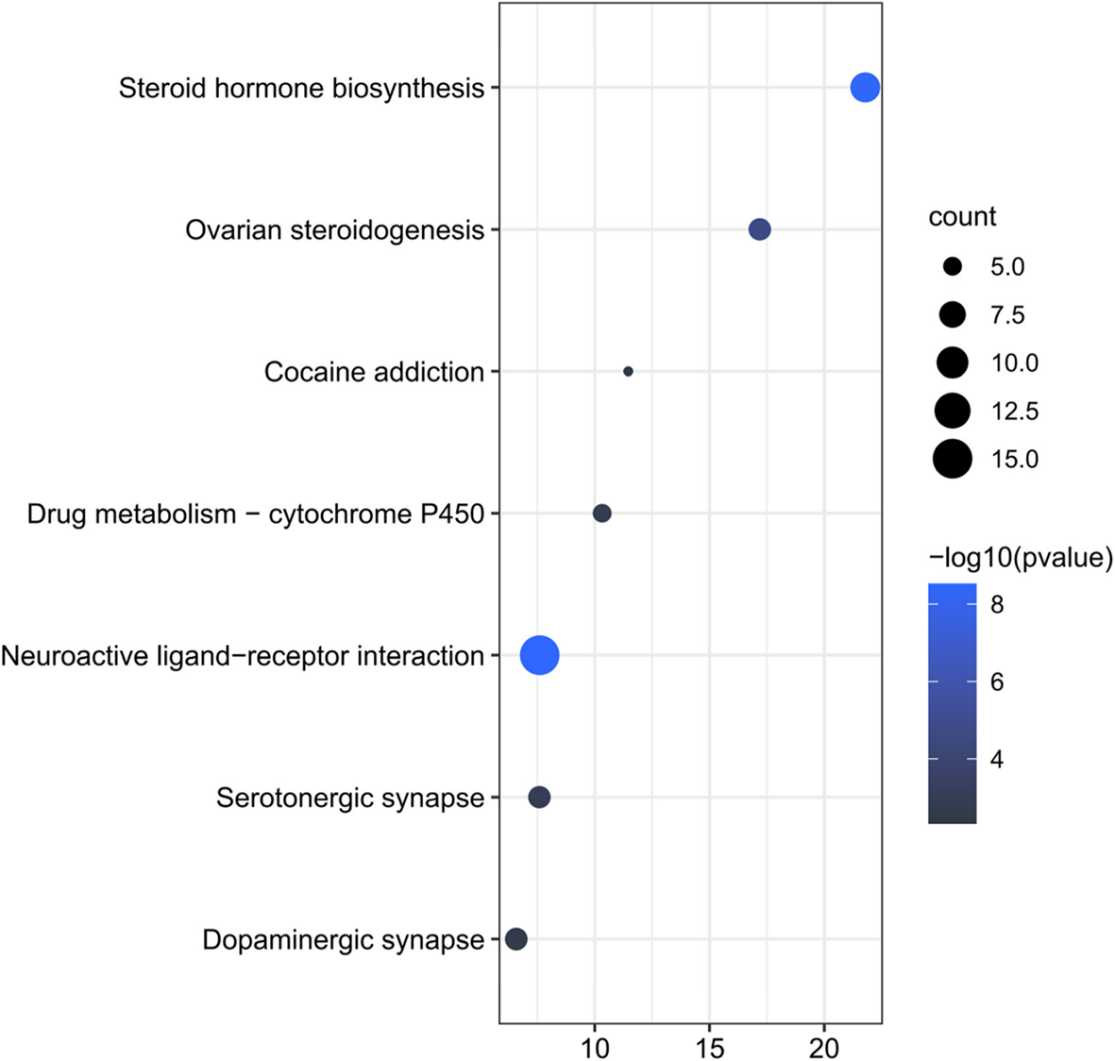
KEGG pathway enrichment analysis of potential targets of orally absorbed compounds from Zishen Yutai pills in POF

Moreover, KEGG pathway enrichment analysis demonstrated that seven pathways (*P* < 0.05) were affected by the compounds absorbed from Zishen Yutai pills in the in vivo POF model, namely steroid hormone biosynthesis, ovarian steroidogenesis, cocaine addiction, cytochrome P450, neuroactive ligand-receptor interaction, serotonergic synapse, and dopaminergic synapse **(**Fig. 6 and Table S4). These pathways were associated with steroid hormone biosynthesis and metabolism (3 pathways), drug metabolism (2 pathways), and signal transduction (2 pathways).

## Discussion

Zishen Yutai pill, a Chinese patent medicine, has been widely used for fertility and reproduction for decades [9, 10]. Although multiple monomeric compounds in the Zishen Yutai pills exerted their therapeutic effects in improving reproduction, the combined activity especially of those absorbed compounds into the blood circulation and their metabolites in vivo was poorly understood. Recently, the Zishen Yutai pills showed therapeutic effects on ovarian dysfunction such as diminished ovarian reserve, primary ovarian insufficiency, and polycystic ovary syndrome [31].

In ovarian dysfunction, POF patients struggle with the loss of gametogenic and steroidogenic functions of the ovary and low probability of pregnancy; it impacts approximately one in 100 women under the age of 40 years; there are currently no effective treatments available [32]. The recent clinical studies were observed that the use of Zishen Yutai pills to increase expression of FSH and LH, and improve hormone disorders in the POF patients [12, 13]. However, the therapeutic mechanism of Zishen Yutai pills in vivo remains obscure.

In this study, we first identified the absorbed compounds of Zishen Yutai pills in POF mice via UHPLC-Q-TOF-MS and employed a network pharmacology–based prediction of the absorbed compound-related gene targets and gene-pathway interactions to interpret the therapeutic mechanism of Zishen Yutai pills in treating POF.

A total of 19 compounds were identified in the POF mice after treatment with Zishen Yutai pills compared to those treated with the solvent. Among these compounds, 6 ingredients were previously identified in methanol extract of Zishen Yutai pills and 13 ingredients were newly identified [13].

We developed the network pharmacology analysis on basis of the chemical profile of Zishen Yutai pills*,* which presented 695 candidate gene targets interacting with 19 potentially active compounds of Zishen Yutai pills. Combining 695 candidate targets and 344 POF-related genes, there were 56 overlapping gene targets of Zishen Yutai pills’ compounds in the treatment of POF. In this absorbed compounds of Zishen Yutai pill-related gene targets, *CYP19A1*, *AKR1C3*, *ESR1*, *AR*, and *SRD5A2* were selected as potential core genes with a higher degree of probability compared to other gene targets.

CYP19A1 is the key steroidogenic enzyme that transforms androstenedione to estrone and testosterone to E2 in granulosa cells [33, 34]. Inhibition of CYP19A1 could reduce the steroidogenic capacity of antral follicles [35]. AKR1C3 is one of four members of the AKR superfamily among the 17β-hydroxysteroid dehydrogenase (17β-HSD) type 5 (HSD17B5) [36, 37]. As a 17β-HSD type 5, AKR1C3 regulates the bioactivity and biosynthesis of steroids by the different extent of 3-keto-, 17-keto-, and 20-ketosteroid reduction [38]. It also acts as a co-activator of AR in its moonlighting function [39].

ESR1 regulates steroidogenesis in theca cells and acts on estrogenic chemicals by stimulating the downstream signaling pathways of the ovary to participate in follicle genesis, growth, and maturation [33, 40]. It has also been demonstrated that ESR1 mutations have a strong association with POF development [41].

AR is primarily found in the granulosa cells and oocytes of the ovary and is involved in maintaining female fertility by optimizing follicular growth, final follicle development, and ovulation [42]. From the results of AR-deficient female mice, a loss of AR protein showed an early decrease in follicle numbers and increased atresia resulting in POF development, indicating that AR may influence follicle atresia by affecting somatic cells apoptosis or oocyte degeneration [43]. SRD5A2 is reported to affect steroid hormones by conversion of testosterone to dihydrotestosterone, a more potent androgen than testosterone. It has also been demonstrated that SRD5A2 plays a key role in androgen metabolism [44].

A total of 56 overlapped predicted targets have been found to be associated with the Zishen Yutai pills in the POF treatment. These target points might be enriched by hormone metabolic and biosynthetic processes and chemical synaptic transmission. Most of the enriched signaling pathways were associated with neuroactive ligand-receptor interaction, steroid hormone biosynthesis, and ovarian steroidogenesis.

Among the 56 overlapped predicted targets, 15 genes were involved in neuroactive ligand-receptor interaction. Most of the 15 genes related to the rhodopsin-like receptors, one of the largest families in the G-protein-coupled receptors (GPCRs). These rhodopsin-like GPCRs include hormones, neuropeptides, neurotransmitters, and light receptors, transducing extracellular signals into specifically targeted cells [45]. This predicted result indicated that Zishen Yutai pills might affect POF by regulating GPCR-mediated cellular activities.

Three classes of steroid hormones—estrogens, progesterone, and androgens—are regarded as part of the ovary reproductive unit. These steroid hormones can be synthesized by the enzymes in the distinctive steroid-producing cells of the ovary. The enzymes are located in the mitochondria and endoplasmic reticulum and include five hydroxylases, two dehydrogenases, a reductase, and an aromatase [46]. Among these important enzymes, 17β-HSD and CYP19 were predicted in our KEGG enrichment results. 17β-HSD converts androstenedione to testosterone and estrone to E2 in granulosa cells. In the presence of CYP19, the converted testosterone could transform to E2 in the granulosa cells and then release into circulation for further maturation and maintenance of the reproductive system.

Granulosa and theca cells work together to organize the biosynthesis of ovarian steroids. In the results of the predicted pathway, six potential targets, INSR, HSD17B2, AKR1C3 (HSD17B5), CYP19A1, CYP17A1, and IGF1R might be involved. INSR and IGF1R are important for follicular development, ovulation, and luteinization, as demonstrated by the conditional knockout of *INSR* and *IGF1R* mice [47]. CYP17A1 works only in theca cells to transform pregnenolone to androgens, providing the substrate for CYP19 to convert to estrogens [46]. HSD17B2, AKR1C3 (HSD17B5), and CYP19A1 are involved not only in steroid hormone biosynthesis but also in ovarian steroidogenesis.

Because estrogen is essential for reproduction, estrogen therapy has become the mainstay of POF treatment [48]. However, limited data is available on the efficacy and safety of long-term use.

Therefore, our findings suggested that the Zishen Yutai pills treated POF primarily by regulating neuroactive ligand-receptor interactions, steroid hormone biosynthesis, and ovarian steroidogenesis resulting in improved levels and bioactivity of E2 and clinical symptoms of the hormone disorders in patients with POF [11, 12].

## Conclusions

In conclusion, our virtual screening results suggested that Zishen Yutai pills treated POF primarily by regulating neuroactive ligand-receptor interactions, steroid hormone biosynthesis, and ovarian steroidogenesis. The combining the absorbed compounds of Zishen Yutai pills in vivo in the POF models and network pharmacology prediction might offer a tool to characterize the mechanism of Zishen Yutai pills in the POF. However, our study has limitations since it has not been validated by additional animal experiments and clinical trials. The animal study for confirming the results of network pharmacology would be investigated in a future study.

## Supplementary Information

**Additional file 1: Table S1.** The edge count of the compound-target-pathway network for the absorbed compounds of Zishen Yutai pills by orally administered in a POF mouse model. **Table S2.** The tabular form of PPI result. **Table S3.** The target degree for PPI network results. **Table S4.** KEGG Pathway analysis of proteins regulated by the absorbed compounds from Zishen Yutai pills in the POF treatment.

## Data Availability

The dataset(s) supporting the conclusions of this article is (are) included within the article (and its additional file(s)).

## Abbreviations

ABCB1: ATP Binding Cassette Subfamily B Member 1
ACE: Angiotensin I Converting Enzyme
ACVR2B: Activin A Receptor Type 2B
AKR: Aldo-Keto Reductase Family
AKR1C2: Aldo-Keto Reductase Family 1 Member C2
AKR1C3: Aldo-Keto Reductase Family 1 Member C3
AMH: Anti-Müllerian hormone
APBA3: Amyloid Beta Precursor Protein Binding Family A Member 3
AR: Androgen Receptor
BP: Biological processes
CC: Cellular compounds
CCR3: C-C Motif Chemokine Receptor 3
CHFR: Checkpoint With Forkhead And Ring Finger Domains
CHRNA4: Cholinergic Receptor Nicotinic Alpha 4 Subunit
CHRNA7: Cholinergic Receptor Nicotinic Alpha 7 Subunit
CITED2: Cbp/P300 Interacting Transactivator With Glu/Asp Rich Carboxy-Terminal Domain 2
CMC-Na: Sodium Carboxymethyl Cellulose
CNR1: Cannabinoid Receptor 1
CRHR1: Corticotropin Releasing Hormone Receptor 1
CYP2C19: Cytochrome P450 Family 2 Subfamily C Member 19
CYP17A1: Cytochrome P450 Family 17 Subfamily A Member 1
CYP19: Cytochrome P450 Family 19
CYP19A1: Cytochrome P450 Family 19 Subfamily A Member 1
DRD2: Dopamine Receptor D2
DRD3: Dopamine Receptor D3
ESI: Electrospray ionization
ESR1: Estrogen Receptor 1
ESR2: Estrogen Receptor 2
E2: Estradiol
FGFR1: Fibroblast Growth Factor Receptor 1
FSH: Follicle-stimulating hormone
F2: Coagulation Factor II, Thrombin
GABBR2: Gamma-Aminobutyric Acid Type B Receptor Subunit 2
GAD: Genetic Association Database
GO: Go enrichment analysis gene ontology
GPCRs: G-protein-coupled receptors
GSTT1: Glutathione S-Transferase Theta 1
HSD17B2: Hydroxysteroid 17-Beta Dehydrogenase 2
HSD17B3: Hydroxysteroid 17-Beta Dehydrogenase 3
HSD17B4: Hydroxysteroid 17-Beta Dehydrogenase 4
HSD17B5: 17β-hydroxysteroid dehydrogenase type 5
HTR1A: 5-Hydroxytryptamine Receptor 1A
HTR2A: 5-Hydroxytryptamine Receptor 2A
IGFBP2: Insulin Like Growth Factor Binding Protein 2
IGF1R: Insulin Like Growth Factor 1 Receptor
IL6: Interleukin 6
INSR: Insulin Receptor
IVF: In vitro fertilization
KEGG: Kyoto Encyclopedia of Genes and Genomes
LH: Luteinizing hormone
MAOA: Monoamine Oxidase A
MAOB: Monoamine Oxidase B
MAP K14: Mitogen-Activated Protein Kinase 14
MF: Molecular functions
NR3C1: Nuclear Receptor Subfamily 3 Group C Member 1
OMIM: Online Mendelian Inheritance in Man Database
OPRD1: Opioid Receptor Delta 1
OPRK1: Opioid Receptor Kappa 1
OPRM1: Opioid Receptor Mu 1
PCSK9: Proprotein Convertase Subtilisin/Kexin Type 9
PGR: Progesterone Receptor
PharmGkb: Pharmacogenomics Knowledge Base
POF: Premature ovarian failure
PPARG: Peroxisome Proliferator Activated Receptor Gamma
PPI: Protein-protein interaction
PTPN11: Protein Tyrosine Phosphatase Non-Receptor Type 11
SERPINE1: Serpin Family E Member 1
SHBG: Sex Hormone Binding Globulin
SLC6A3: Solute Carrier Family 6 Member 3
SLC6A4: Solute Carrier Family 6 Member 4
SRD5A1: Steroid 5 Alpha-Reductase 1
SRD5A2: Steroid 5 Alpha-Reductase 2
SREBF2: Sterol Regulatory Element Binding Transcription Factor 2
SSTR3: Somatostatin Receptor 3
TCM: Traditional Chinese medicine
TCMSP: Traditional Chinese Medicine Systems Pharmacology Database
TNF: Tumor Necrosis Factor
TTD: Therapeutic Target Database
UGT2B7: UDP Glucuronosyltransferase Family 2 Member B7
UHPLC-Q-TOF-MS: Ultra-high performance liquid chromatography-quadrupole time-of-flight mass spectrometry
VDR: Vitamin D Receptor
ZSYT: Zishen Yutai pills
17β-HSD: 17β-hydroxysteroid dehydrogenase
