# Supplementary material for: Mechanisms of action of Zishen Yutai pills in treating premature ovarian failure determined by integrating UHPLC-Q-TOF-MS and network pharmacology analysis
Source: BMC Complement Med Ther. 2022 Oct 26;22:281. doi: 10.1186/s12906-022-03763-2 (PMC9597968; doi:10.1186/s12906-022-03763-2)
Supplement: Supplementary file 1 — Additional file 1: Table S1. The edge count of the compound-target-pathway network for the absorbed compounds of Zishen Yutai pills by orally administered in a POF mouse model. Table S2. The tabular form of PPI result. Table S3. The target degree for PPI network results. Table S4. KEGG Pathway analysis of proteins regulated by the absorbed compounds from Zishen Yutai pills in the POF treatment. [file 12906_2022_3763_MOESM1_ESM.docx]

**Supplementary Material**

**Table S1** The edge count of the compound-target-pathway network for the absorbed compounds of Zishen Yutai pills by orally administered in a POF mouse model.

| **node** | **type** | **edge count** |
| --- | --- | --- |
| ABCB1 | Target | 1 |
| ACE | Target | 2 |
| ACVR2B | Target | 2 |
| AKR1C2 | Target | 3 |
| AKR1C3 | Target | 5 |
| APBA3 | Target | 2 |
| AR | Target | 10 |
| CCR3 | Target | 1 |
| CHFR | Target | 1 |
| CHRNA4 | Target | 2 |
| CHRNA7 | Target | 2 |
| CITED2 | Target | 6 |
| CNR1 | Target | 6 |
| CRHR1 | Target | 3 |
| CYP17A1 | Target | 9 |
| CYP19A1 | Target | 10 |
| CYP2C19 | Target | 5 |
| DRD2 | Target | 5 |
| DRD3 | Target | 4 |
| ESR1 | Target | 6 |
| ESR2 | Target | 7 |
| F2 | Target | 5 |
| FGFR1 | Target | 1 |
| GABBR2 | Target | 3 |
| GSTT1 | Target | 2 |
| HSD17B2 | Target | 4 |
| HSD17B3 | Target | 3 |
| HSD17B4 | Target | 5 |
| HTR1A | Target | 3 |
| HTR2A | Target | 3 |
| IGF1R | Target | 3 |
| IGFBP2 | Target | 1 |
| IL6 | Target | 3 |
| INSR | Target | 2 |
| MAOA | Target | 8 |
| MAOB | Target | 8 |
| MAPK14 | Target | 6 |
| NR3C1 | Target | 8 |
| OPRD1 | Target | 3 |
| OPRK1 | Target | 3 |
| OPRM1 | Target | 3 |
| PCSK9 | Target | 2 |
| PGR | Target | 7 |
| PPARG | Target | 5 |
| PTPN11 | Target | 5 |
| SERPINE1 | Target | 1 |
| SHBG | Target | 7 |
| SLC6A3 | Target | 6 |
| SLC6A4 | Target | 5 |
| SRD5A1 | Target | 2 |
| SRD5A2 | Target | 10 |
| SREBF2 | Target | 1 |
| SSTR3 | Target | 2 |
| TNF | Target | 2 |
| UGT2B7 | Target | 3 |
| VDR | Target | 2 |
| Benzophenone | Compound | 4 |
| 2-[[(3Î±,5Î²)-3-Hydroxy-7,24-dioxocholan-24-yl]amino]ethanesulfonic acid | Compound | 4 |
| Taurocholic acid | Compound | 6 |
| Cortisol | Compound | 21 |
| Corticosterone | Compound | 21 |
| (3α,9ξ,14ξ,16α)-17-Hydroxy-16-methyl-20-oxopregn-5-en-3-yl acetate | Compound | 23 |
| Ethyl N-[(13E,15R)-15-hydroxy-1,9-dioxoprost-13-en-1-yl]glycinate | Compound | 2 |
| 1-[(2R,4aS,4bS,6aS,6bS,9aR,10aS,10bR,12aS)-2-Hydroxy-4a,6a,8,8-tetramethylhexadecahydro-6bH-naphtho[2',1':4,5]indeno[1,2-d][1,3]dioxol-6b-yl]ethanone | Compound | 18 |
| 3-(2-Furylmethyl)-10-methyl-2-(3-nitrophenyl)pyrimido[4,5-b]quinoline-4,5(3H,10H)-dione | Compound | 4 |
| (3β,5α)-16-Methyl-20-oxopregn-16-en-3-yl acetate | Compound | 14 |
| cholic acid | Compound | 8 |
| Tetradecyl (βS)-β-hydroxy-N-(phenylacetyl)-D-phenylalaninate | Compound | 1 |
| 3,5-Pyridinedicarboxylic acid, 1,4-dihydro-2,6-dimethyl-4-[3-[4-(4-morpholinylsulfonyl)phenyl]-1-phenyl-1H-pyrazol-4-yl]-, 3,5-bis(2-methylpropyl) ester | Compound | 8 |
| Betaine | Compound | 2 |
| L-Pyroglutmaic acid | Compound | 2 |
| Isoleucine | Compound | 2 |
| Vernolic acid | Compound | 10 |
| Trichosanic acid | Compound | 11 |
| linolenic acid | Compound | 17 |
| Neuroactive ligand-receptor interaction | Pathway | 15 |
| Steroid hormone biosynthesis | Pathway | 9 |
| Ovarian steroidogenesis | Pathway | 6 |
| Serotonergic synapse | Pathway | 6 |
| Drug metabolism - cytochrome P450 | Pathway | 5 |
| Dopaminergic synapse | Pathway | 6 |
| Cocaine addiction | Pathway | 4 |

**Table S2** The tabular form of PPI result.

| **node1** | **node2** | **node1 accession** | **node2 accession** | **score** |
| --- | --- | --- | --- | --- |
| ABCB1 | CYP2C19 | ENSP00000478255 | ENSP00000360372 | 0.767 |
| ACE | IL6 | ENSP00000290866 | ENSP00000385675 | 0.706 |
| ACE | PPARG | ENSP00000290866 | ENSP00000287820 | 0.785 |
| ACE | SERPINE1 | ENSP00000290866 | ENSP00000223095 | 0.728 |
| ACE | TNF | ENSP00000290866 | ENSP00000398698 | 0.707 |
| AKR1C2 | AKR1C3 | ENSP00000370129 | ENSP00000369927 | 0.978 |
| AKR1C2 | SRD5A1 | ENSP00000370129 | ENSP00000274192 | 0.967 |
| AKR1C2 | SRD5A2 | ENSP00000370129 | ENSP00000477587 | 0.965 |
| AKR1C2 | UGT2B7 | ENSP00000370129 | ENSP00000304811 | 0.739 |
| AKR1C3 | AKR1C2 | ENSP00000369927 | ENSP00000370129 | 0.978 |
| AKR1C3 | AR | ENSP00000369927 | ENSP00000363822 | 0.749 |
| AKR1C3 | CYP17A1 | ENSP00000369927 | ENSP00000358903 | 0.968 |
| AKR1C3 | CYP19A1 | ENSP00000369927 | ENSP00000379683 | 0.955 |
| AKR1C3 | HSD17B2 | ENSP00000369927 | ENSP00000199936 | 0.929 |
| AKR1C3 | HSD17B3 | ENSP00000369927 | ENSP00000364412 | 0.939 |
| AKR1C3 | SRD5A1 | ENSP00000369927 | ENSP00000274192 | 0.975 |
| AKR1C3 | SRD5A2 | ENSP00000369927 | ENSP00000477587 | 0.967 |
| AKR1C3 | UGT2B7 | ENSP00000369927 | ENSP00000304811 | 0.758 |
| AR | AKR1C3 | ENSP00000363822 | ENSP00000369927 | 0.749 |
| AR | CYP17A1 | ENSP00000363822 | ENSP00000358903 | 0.819 |
| AR | CYP19A1 | ENSP00000363822 | ENSP00000379683 | 0.806 |
| AR | IGF1R | ENSP00000363822 | ENSP00000268035 | 0.742 |
| AR | MAPK14 | ENSP00000363822 | ENSP00000229795 | 0.93 |
| AR | NR3C1 | ENSP00000363822 | ENSP00000231509 | 0.93 |
| AR | SHBG | ENSP00000363822 | ENSP00000369816 | 0.922 |
| AR | SRD5A2 | ENSP00000363822 | ENSP00000477587 | 0.807 |
| CCR3 | F2 | ENSP00000441600 | ENSP00000308541 | 0.909 |
| CHRNA4 | CHRNA7 | ENSP00000359285 | ENSP00000407546 | 0.928 |
| CHRNA7 | CHRNA4 | ENSP00000407546 | ENSP00000359285 | 0.928 |
| CITED2 | HSD17B4 | ENSP00000444198 | ENSP00000420914 | 0.9 |
| CNR1 | DRD2 | ENSP00000358513 | ENSP00000354859 | 0.998 |
| CNR1 | PPARG | ENSP00000358513 | ENSP00000287820 | 0.714 |
| CRHR1 | HTR1A | ENSP00000381333 | ENSP00000316244 | 0.721 |
| CRHR1 | HTR2A | ENSP00000381333 | ENSP00000437737 | 0.729 |
| CRHR1 | NR3C1 | ENSP00000381333 | ENSP00000231509 | 0.729 |
| CRHR1 | SLC6A4 | ENSP00000381333 | ENSP00000261707 | 0.803 |
| CYP17A1 | AKR1C3 | ENSP00000358903 | ENSP00000369927 | 0.968 |
| CYP17A1 | AR | ENSP00000358903 | ENSP00000363822 | 0.819 |
| CYP17A1 | CYP19A1 | ENSP00000358903 | ENSP00000379683 | 0.951 |
| CYP17A1 | HSD17B2 | ENSP00000358903 | ENSP00000199936 | 0.961 |
| CYP17A1 | HSD17B3 | ENSP00000358903 | ENSP00000364412 | 0.985 |
| CYP17A1 | SRD5A1 | ENSP00000358903 | ENSP00000274192 | 0.979 |
| CYP17A1 | SRD5A2 | ENSP00000358903 | ENSP00000477587 | 0.976 |
| CYP19A1 | AKR1C3 | ENSP00000379683 | ENSP00000369927 | 0.955 |
| CYP19A1 | AR | ENSP00000379683 | ENSP00000363822 | 0.806 |
| CYP19A1 | CYP17A1 | ENSP00000379683 | ENSP00000358903 | 0.951 |
| CYP19A1 | ESR1 | ENSP00000379683 | ENSP00000405330 | 0.922 |
| CYP19A1 | ESR2 | ENSP00000379683 | ENSP00000343925 | 0.885 |
| CYP19A1 | HSD17B2 | ENSP00000379683 | ENSP00000199936 | 0.965 |
| CYP19A1 | HSD17B3 | ENSP00000379683 | ENSP00000364412 | 0.963 |
| CYP19A1 | PGR | ENSP00000379683 | ENSP00000325120 | 0.843 |
| CYP19A1 | SHBG | ENSP00000379683 | ENSP00000369816 | 0.733 |
| CYP19A1 | SRD5A1 | ENSP00000379683 | ENSP00000274192 | 0.969 |
| CYP19A1 | SRD5A2 | ENSP00000379683 | ENSP00000477587 | 0.964 |
| CYP19A1 | UGT2B7 | ENSP00000379683 | ENSP00000304811 | 0.733 |
| CYP2C19 | ABCB1 | ENSP00000360372 | ENSP00000478255 | 0.767 |
| CYP2C19 | MAOA | ENSP00000360372 | ENSP00000340684 | 0.932 |
| CYP2C19 | MAOB | ENSP00000360372 | ENSP00000367309 | 0.928 |
| CYP2C19 | UGT2B7 | ENSP00000360372 | ENSP00000304811 | 0.74 |
| DRD2 | CNR1 | ENSP00000354859 | ENSP00000358513 | 0.998 |
| DRD2 | MAOA | ENSP00000354859 | ENSP00000340684 | 0.757 |
| DRD2 | SLC6A3 | ENSP00000354859 | ENSP00000270349 | 0.997 |
| DRD2 | SLC6A4 | ENSP00000354859 | ENSP00000261707 | 0.855 |
| DRD3 | SLC6A3 | ENSP00000373169 | ENSP00000270349 | 0.763 |
| ESR1 | CYP19A1 | ENSP00000405330 | ENSP00000379683 | 0.922 |
| ESR1 | ESR2 | ENSP00000405330 | ENSP00000343925 | 0.987 |
| ESR1 | IGF1R | ENSP00000405330 | ENSP00000268035 | 0.999 |
| ESR1 | MAPK14 | ENSP00000405330 | ENSP00000229795 | 0.956 |
| ESR1 | NR3C1 | ENSP00000405330 | ENSP00000231509 | 0.93 |
| ESR1 | PGR | ENSP00000405330 | ENSP00000325120 | 0.952 |
| ESR1 | PTPN11 | ENSP00000405330 | ENSP00000340944 | 0.9 |
| ESR1 | SHBG | ENSP00000405330 | ENSP00000369816 | 0.835 |
| ESR2 | CYP19A1 | ENSP00000343925 | ENSP00000379683 | 0.885 |
| ESR2 | ESR1 | ENSP00000343925 | ENSP00000405330 | 0.987 |
| ESR2 | MAPK14 | ENSP00000343925 | ENSP00000229795 | 0.855 |
| F2 | CCR3 | ENSP00000308541 | ENSP00000441600 | 0.909 |
| F2 | SERPINE1 | ENSP00000308541 | ENSP00000223095 | 0.901 |
| FGFR1 | HTR1A | ENSP00000393312 | ENSP00000316244 | 0.993 |
| FGFR1 | PTPN11 | ENSP00000393312 | ENSP00000340944 | 0.839 |
| HSD17B2 | AKR1C3 | ENSP00000199936 | ENSP00000369927 | 0.929 |
| HSD17B2 | CYP17A1 | ENSP00000199936 | ENSP00000358903 | 0.961 |
| HSD17B2 | CYP19A1 | ENSP00000199936 | ENSP00000379683 | 0.965 |
| HSD17B2 | HSD17B3 | ENSP00000199936 | ENSP00000364412 | 0.981 |
| HSD17B2 | HSD17B4 | ENSP00000199936 | ENSP00000420914 | 0.836 |
| HSD17B2 | SRD5A1 | ENSP00000199936 | ENSP00000274192 | 0.964 |
| HSD17B2 | SRD5A2 | ENSP00000199936 | ENSP00000477587 | 0.958 |
| HSD17B3 | AKR1C3 | ENSP00000364412 | ENSP00000369927 | 0.939 |
| HSD17B3 | CYP17A1 | ENSP00000364412 | ENSP00000358903 | 0.985 |
| HSD17B3 | CYP19A1 | ENSP00000364412 | ENSP00000379683 | 0.963 |
| HSD17B3 | HSD17B2 | ENSP00000364412 | ENSP00000199936 | 0.981 |
| HSD17B3 | HSD17B4 | ENSP00000364412 | ENSP00000420914 | 0.91 |
| HSD17B3 | SRD5A1 | ENSP00000364412 | ENSP00000274192 | 0.98 |
| HSD17B3 | SRD5A2 | ENSP00000364412 | ENSP00000477587 | 0.98 |
| HSD17B4 | CITED2 | ENSP00000420914 | ENSP00000444198 | 0.9 |
| HSD17B4 | HSD17B2 | ENSP00000420914 | ENSP00000199936 | 0.836 |
| HSD17B4 | HSD17B3 | ENSP00000420914 | ENSP00000364412 | 0.91 |
| HTR1A | CRHR1 | ENSP00000316244 | ENSP00000381333 | 0.721 |
| HTR1A | FGFR1 | ENSP00000316244 | ENSP00000393312 | 0.993 |
| HTR1A | MAOA | ENSP00000316244 | ENSP00000340684 | 0.772 |
| HTR1A | SLC6A3 | ENSP00000316244 | ENSP00000270349 | 0.777 |
| HTR1A | SLC6A4 | ENSP00000316244 | ENSP00000261707 | 0.982 |
| HTR2A | CRHR1 | ENSP00000437737 | ENSP00000381333 | 0.729 |
| HTR2A | MAOA | ENSP00000437737 | ENSP00000340684 | 0.782 |
| HTR2A | SLC6A3 | ENSP00000437737 | ENSP00000270349 | 0.718 |
| HTR2A | SLC6A4 | ENSP00000437737 | ENSP00000261707 | 0.914 |
| IGF1R | AR | ENSP00000268035 | ENSP00000363822 | 0.742 |
| IGF1R | ESR1 | ENSP00000268035 | ENSP00000405330 | 0.999 |
| IGF1R | IGFBP2 | ENSP00000268035 | ENSP00000233809 | 0.74 |
| IGF1R | INSR | ENSP00000268035 | ENSP00000303830 | 0.971 |
| IGF1R | PTPN11 | ENSP00000268035 | ENSP00000340944 | 0.999 |
| IGFBP2 | IGF1R | ENSP00000233809 | ENSP00000268035 | 0.74 |
| IL6 | ACE | ENSP00000385675 | ENSP00000290866 | 0.706 |
| IL6 | MAPK14 | ENSP00000385675 | ENSP00000229795 | 0.758 |
| IL6 | PPARG | ENSP00000385675 | ENSP00000287820 | 0.792 |
| IL6 | PTPN11 | ENSP00000385675 | ENSP00000340944 | 0.958 |
| IL6 | SERPINE1 | ENSP00000385675 | ENSP00000223095 | 0.827 |
| IL6 | TNF | ENSP00000385675 | ENSP00000398698 | 0.994 |
| INSR | IGF1R | ENSP00000303830 | ENSP00000268035 | 0.971 |
| INSR | PTPN11 | ENSP00000303830 | ENSP00000340944 | 0.988 |
| MAOA | CYP2C19 | ENSP00000340684 | ENSP00000360372 | 0.932 |
| MAOA | DRD2 | ENSP00000340684 | ENSP00000354859 | 0.757 |
| MAOA | HTR1A | ENSP00000340684 | ENSP00000316244 | 0.772 |
| MAOA | HTR2A | ENSP00000340684 | ENSP00000437737 | 0.782 |
| MAOA | MAOB | ENSP00000340684 | ENSP00000367309 | 0.88 |
| MAOA | SLC6A3 | ENSP00000340684 | ENSP00000270349 | 0.804 |
| MAOA | SLC6A4 | ENSP00000340684 | ENSP00000261707 | 0.887 |
| MAOB | CYP2C19 | ENSP00000367309 | ENSP00000360372 | 0.928 |
| MAOB | MAOA | ENSP00000367309 | ENSP00000340684 | 0.88 |
| MAOB | SLC6A3 | ENSP00000367309 | ENSP00000270349 | 0.785 |
| MAPK14 | AR | ENSP00000229795 | ENSP00000363822 | 0.93 |
| MAPK14 | ESR1 | ENSP00000229795 | ENSP00000405330 | 0.956 |
| MAPK14 | ESR2 | ENSP00000229795 | ENSP00000343925 | 0.855 |
| MAPK14 | IL6 | ENSP00000229795 | ENSP00000385675 | 0.758 |
| MAPK14 | NR3C1 | ENSP00000229795 | ENSP00000231509 | 0.935 |
| MAPK14 | PGR | ENSP00000229795 | ENSP00000325120 | 0.85 |
| MAPK14 | TNF | ENSP00000229795 | ENSP00000398698 | 0.975 |
| NR3C1 | AR | ENSP00000231509 | ENSP00000363822 | 0.93 |
| NR3C1 | CRHR1 | ENSP00000231509 | ENSP00000381333 | 0.729 |
| NR3C1 | ESR1 | ENSP00000231509 | ENSP00000405330 | 0.93 |
| NR3C1 | MAPK14 | ENSP00000231509 | ENSP00000229795 | 0.935 |
| NR3C1 | TNF | ENSP00000231509 | ENSP00000398698 | 0.96 |
| PCSK9 | SREBF2 | ENSP00000303208 | ENSP00000354476 | 0.757 |
| PGR | CYP19A1 | ENSP00000325120 | ENSP00000379683 | 0.843 |
| PGR | ESR1 | ENSP00000325120 | ENSP00000405330 | 0.952 |
| PGR | MAPK14 | ENSP00000325120 | ENSP00000229795 | 0.85 |
| PPARG | ACE | ENSP00000287820 | ENSP00000290866 | 0.785 |
| PPARG | CNR1 | ENSP00000287820 | ENSP00000358513 | 0.714 |
| PPARG | IL6 | ENSP00000287820 | ENSP00000385675 | 0.792 |
| PPARG | SREBF2 | ENSP00000287820 | ENSP00000354476 | 0.964 |
| PPARG | TNF | ENSP00000287820 | ENSP00000398698 | 0.979 |
| PTPN11 | ESR1 | ENSP00000340944 | ENSP00000405330 | 0.9 |
| PTPN11 | FGFR1 | ENSP00000340944 | ENSP00000393312 | 0.839 |
| PTPN11 | IGF1R | ENSP00000340944 | ENSP00000268035 | 0.999 |
| PTPN11 | IL6 | ENSP00000340944 | ENSP00000385675 | 0.958 |
| PTPN11 | INSR | ENSP00000340944 | ENSP00000303830 | 0.988 |
| SERPINE1 | ACE | ENSP00000223095 | ENSP00000290866 | 0.728 |
| SERPINE1 | F2 | ENSP00000223095 | ENSP00000308541 | 0.901 |
| SERPINE1 | IL6 | ENSP00000223095 | ENSP00000385675 | 0.827 |
| SERPINE1 | TNF | ENSP00000223095 | ENSP00000398698 | 0.762 |
| SHBG | AR | ENSP00000369816 | ENSP00000363822 | 0.922 |
| SHBG | CYP19A1 | ENSP00000369816 | ENSP00000379683 | 0.733 |
| SHBG | ESR1 | ENSP00000369816 | ENSP00000405330 | 0.835 |
| SLC6A3 | DRD2 | ENSP00000270349 | ENSP00000354859 | 0.997 |
| SLC6A3 | DRD3 | ENSP00000270349 | ENSP00000373169 | 0.763 |
| SLC6A3 | HTR1A | ENSP00000270349 | ENSP00000316244 | 0.777 |
| SLC6A3 | HTR2A | ENSP00000270349 | ENSP00000437737 | 0.718 |
| SLC6A3 | MAOA | ENSP00000270349 | ENSP00000340684 | 0.804 |
| SLC6A3 | MAOB | ENSP00000270349 | ENSP00000367309 | 0.785 |
| SLC6A4 | CRHR1 | ENSP00000261707 | ENSP00000381333 | 0.803 |
| SLC6A4 | DRD2 | ENSP00000261707 | ENSP00000354859 | 0.855 |
| SLC6A4 | HTR1A | ENSP00000261707 | ENSP00000316244 | 0.982 |
| SLC6A4 | HTR2A | ENSP00000261707 | ENSP00000437737 | 0.914 |
| SLC6A4 | MAOA | ENSP00000261707 | ENSP00000340684 | 0.887 |
| SRD5A1 | AKR1C2 | ENSP00000274192 | ENSP00000370129 | 0.967 |
| SRD5A1 | AKR1C3 | ENSP00000274192 | ENSP00000369927 | 0.975 |
| SRD5A1 | CYP17A1 | ENSP00000274192 | ENSP00000358903 | 0.979 |
| SRD5A1 | CYP19A1 | ENSP00000274192 | ENSP00000379683 | 0.969 |
| SRD5A1 | HSD17B2 | ENSP00000274192 | ENSP00000199936 | 0.964 |
| SRD5A1 | HSD17B3 | ENSP00000274192 | ENSP00000364412 | 0.98 |
| SRD5A1 | SRD5A2 | ENSP00000274192 | ENSP00000477587 | 0.818 |
| SRD5A2 | AKR1C2 | ENSP00000477587 | ENSP00000370129 | 0.965 |
| SRD5A2 | AKR1C3 | ENSP00000477587 | ENSP00000369927 | 0.967 |
| SRD5A2 | AR | ENSP00000477587 | ENSP00000363822 | 0.807 |
| SRD5A2 | CYP17A1 | ENSP00000477587 | ENSP00000358903 | 0.976 |
| SRD5A2 | CYP19A1 | ENSP00000477587 | ENSP00000379683 | 0.964 |
| SRD5A2 | HSD17B2 | ENSP00000477587 | ENSP00000199936 | 0.958 |
| SRD5A2 | HSD17B3 | ENSP00000477587 | ENSP00000364412 | 0.98 |
| SRD5A2 | SRD5A1 | ENSP00000477587 | ENSP00000274192 | 0.818 |
| SREBF2 | PCSK9 | ENSP00000354476 | ENSP00000303208 | 0.757 |
| SREBF2 | PPARG | ENSP00000354476 | ENSP00000287820 | 0.964 |
| TNF | ACE | ENSP00000398698 | ENSP00000290866 | 0.707 |
| TNF | IL6 | ENSP00000398698 | ENSP00000385675 | 0.994 |
| TNF | MAPK14 | ENSP00000398698 | ENSP00000229795 | 0.975 |
| TNF | NR3C1 | ENSP00000398698 | ENSP00000231509 | 0.96 |
| TNF | PPARG | ENSP00000398698 | ENSP00000287820 | 0.979 |
| TNF | SERPINE1 | ENSP00000398698 | ENSP00000223095 | 0.762 |
| TNF | VDR | ENSP00000398698 | ENSP00000447173 | 0.718 |
| UGT2B7 | AKR1C2 | ENSP00000304811 | ENSP00000370129 | 0.739 |
| UGT2B7 | AKR1C3 | ENSP00000304811 | ENSP00000369927 | 0.758 |
| UGT2B7 | CYP19A1 | ENSP00000304811 | ENSP00000379683 | 0.733 |
| UGT2B7 | CYP2C19 | ENSP00000304811 | ENSP00000360372 | 0.74 |
| VDR | TNF | ENSP00000447173 | ENSP00000398698 | 0.718 |

**Table S3** The target degree for PPI network results.

| **RANK** | **gene name** | **protein name** | **degree** |
| --- | --- | --- | --- |
| 1 | CYP19A1 | Cytochrome P450 Family 19 Subfamily A Member 1 | 12 |
| 2 | AKR1C3 | Aldo-Keto Reductase Family 1 Member C3 | 9 |
| 3 | ESR1 | Estrogen Receptor 1 | 8 |
| 4 | AR | Androgen Receptor | 8 |
| 5 | SRD5A2 | Steroid 5 Alpha-Reductase 2 | 8 |
| 6 | MAOA | Monoamine Oxidase A | 7 |
| 7 | MAPK14 | mitogen-activated protein kinase 14 | 7 |
| 8 | HSD17B3 | Hydroxysteroid 17-Beta Dehydrogenase 3 | 7 |
| 9 | CYP17A1 | Cytochrome P450 Family 17 Subfamily A Member 1 | 7 |
| 10 | HSD17B2 | Hydroxysteroid 17-Beta Dehydrogenase 2 | 7 |
| 11 | SRD5A1 | Steroid 5 Alpha-Reductase 1 | 7 |
| 12 | TNF | Tumor Necrosis Factor | 7 |
| 13 | SLC6A3 | Solute Carrier Family 6 Member 3 | 6 |
| 14 | IL6 | Interleukin 6 | 6 |
| 15 | PTPN11 | Protein Tyrosine Phosphatase Non-Receptor Type 11 | 5 |
| 16 | HTR1A | 5-Hydroxytryptamine Receptor 1A | 5 |
| 17 | SLC6A4 | Solute Carrier Family 6 Member 4 | 5 |
| 18 | IGF1R | Insulin Like Growth Factor 1 Receptor | 5 |
| 19 | NR3C1 | Nuclear Receptor Subfamily 3 Group C Member 1 | 5 |
| 20 | PPARG | Peroxisome Proliferator Activated Receptor Gamma | 5 |
| 21 | HTR2A | 5-Hydroxytryptamine Receptor 2A | 4 |
| 22 | CRHR1 | Corticotropin Releasing Hormone Receptor 1 | 4 |
| 23 | DRD2 | Dopamine Receptor D2 | 4 |
| 24 | UGT2B7 | UDP Glucuronosyltransferase Family 2 Member B7 | 4 |
| 25 | AKR1C2 | Aldo-Keto Reductase Family 1 Member C2 | 4 |
| 26 | SERPINE1 | Serpin Family E Member 1 | 4 |
| 27 | ACE | Angiotensin I Converting Enzyme | 4 |
| 28 | CYP2C19 | Cytochrome P450 Family 2 Subfamily C Member 19 | 4 |
| 29 | MAOB | Monoamine Oxidase B | 3 |
| 30 | ESR2 | Estrogen Receptor 2 | 3 |
| 31 | PGR | Progesterone Receptor | 3 |
| 32 | HSD17B4 | Hydroxysteroid 17-Beta Dehydrogenase 4 | 3 |
| 33 | SHBG | Sex Hormone Binding Globulin | 3 |
| 34 | SREBF2 | Sterol Regulatory Element Binding Transcription Factor 2 | 2 |
| 35 | INSR | Insulin Receptor | 2 |
| 36 | FGFR1 | Fibroblast Growth Factor Receptor 1 | 2 |
| 37 | CNR1 | Cannabinoid Receptor 1 | 2 |
| 38 | F2 | Coagulation Factor II, Thrombin | 2 |
| 39 | VDR | Vitamin D Receptor | 1 |
| 40 | PCSK9 | Proprotein Convertase Subtilisin/Kexin Type 9 | 1 |
| 41 | IGFBP2 | Insulin Like Growth Factor Binding Protein 2 | 1 |
| 42 | DRD3 | Dopamine Receptor D3 | 1 |
| 43 | CITED2 | Cbp/P300 Interacting Transactivator With Glu/Asp Rich Carboxy-Terminal Domain 2 | 1 |
| 44 | CHRNA7 | Cholinergic Receptor Nicotinic Alpha 7 Subunit | 1 |
| 45 | CHRNA4 | Cholinergic Receptor Nicotinic Alpha 4 Subunit | 1 |
| 46 | CCR3 | C-C Motif Chemokine Receptor 3 | 1 |
| 47 | ABCB1 | ATP Binding Cassette Subfamily B Member 1 | 1 |

**Table S4** KEGG Pathway analysis of proteins regulated by the absorbed compounds from Zishen Yutai pills in the POF treatment.

| **pathway ID** | **pathway description** | **count in gene set** | **name of genes** | **P value** | **false discovery rate** |
| --- | --- | --- | --- | --- | --- |
| hsa04080 | Neuroactive ligand-receptor interaction | 15 | OPRD1, GABBR2, CHRNA4, CHRNA7, HTR1A, OPRK1, HTR2A, OPRM1, F2, NR3C1, SSTR3, CRHR1, CNR1, DRD2, DRD3 | 2.99E-09 | 3.04E-07 |
| hsa00140 | Steroid hormone biosynthesis | 9 | SRD5A2, SRD5A1, HSD17B2, AKR1C3, HSD17B3, AKR1C2, CYP19A1, UGT2B7, CYP17A1 | 4.50E-09 | 3.04E-07 |
| hsa04913 | Ovarian steroidogenesis | 6 | INSR, HSD17B2, AKR1C3, CYP19A1, CYP17A1, IGF1R | 2.02E-05 | 9.11E-04 |
| hsa04726 | Serotonergic synapse | 6 | MAOB, MAOA, HTR1A, HTR2A, CYP2C19, SLC6A4 | 9.85E-04 | 0.0331 |
| hsa00982 | Drug metabolism - cytochrome P450 | 5 | MAOB, MAOA, GSTT1, CYP2C19, UGT2B7 | 0.0012 | 0.0331 |
| hsa04728 | Dopaminergic synapse | 6 | MAOB, MAOA, DRD2, MAPK14, DRD3, SLC6A3 | 0.0019 | 0.0419 |
| hsa05030 | Cocaine addiction | 4 | MAOB, MAOA, DRD2, SLC6A3 | 0.0047 | 0.0905 |
